# Identifying candidate drivers of drug response in heterogeneous cancer by mining high throughput genomics data

**DOI:** 10.1186/s12864-016-2942-5

**Published:** 2016-08-15

**Authors:** Sheida Nabavi

**Affiliations:** Computer Science and Engineering Department, Institute for Systems Genomics, University of Connecticut, 371 Fairfield Way, Unit 4155, Storrs, CT 06268 USA

**Keywords:** Integrative analysis, Module network analysis, Gene module, Copy number aberration, Somatic mutation, Gene expression, Serous ovarian carcinoma, Drug resistant

## Abstract

**Background:**

With advances in technologies, huge amounts of multiple types of high-throughput genomics data are available. These data have tremendous potential to identify new and clinically valuable biomarkers to guide the diagnosis, assessment of prognosis, and treatment of complex diseases, such as cancer. Integrating, analyzing, and interpreting big and noisy genomics data to obtain biologically meaningful results, however, remains highly challenging. Mining genomics datasets by utilizing advanced computational methods can help to address these issues.

**Results:**

To facilitate the identification of a short list of biologically meaningful genes as candidate drivers of anti-cancer drug resistance from an enormous amount of heterogeneous data, we employed statistical machine-learning techniques and integrated genomics datasets. We developed a computational method that integrates gene expression, somatic mutation, and copy number aberration data of sensitive and resistant tumors. In this method, an integrative method based on module network analysis is applied to identify potential driver genes. This is followed by cross-validation and a comparison of the results of sensitive and resistance groups to obtain the final list of candidate biomarkers. We applied this method to the ovarian cancer data from the cancer genome atlas. The final result contains biologically relevant genes, such as COL11A1, which has been reported as a cis-platinum resistant biomarker for epithelial ovarian carcinoma in several recent studies.

**Conclusions:**

The described method yields a short list of aberrant genes that also control the expression of their co-regulated genes. The results suggest that the unbiased data driven computational method can identify biologically relevant candidate biomarkers. It can be utilized in a wide range of applications that compare two conditions with highly heterogeneous datasets.

**Electronic supplementary material:**

The online version of this article (doi:10.1186/s12864-016-2942-5) contains supplementary material, which is available to authorized users.

## Background

Cancer is known as a disease of the genome. A cancer genome harbors thousands of genomics aberrations in the form of large segment aberrations and somatic point mutations. Not all of the aberrations, however, have important roles in tumor progression. To understand the mechanism of cancer, biomedical researchers are interested in identifying genomic aberrations that drive cancer progression, which are termed *drivers*. They also are interested in profiling gene expression data to better understand disease pathogenesis. Activation or deactivation of the functional parts of a genome, or genes, determines the pathological states and development of a disease. For example, the over-expression of an oncogene or under-expression of a tumor suppressor gene plays an important role in cancer pathogenesis. Most likely, a driver gene is among the over/under-expressed genes that also have aberrations. It is reasonable to consider that an over/under-expressed driver gene has a footprint in a genome in the form of an aberration that can be used as a biomarker [1, 2]. It is hypothesized that driver genes involved in resistance likely have aberrant copy numbers and/or mutations and that the expression patterns of these genes match the mutation and copy number patterns. The important role of genomics aberrations, incorporated with gene expression profiles in disease progression, has motivated several studies [2, 3] to integrate copy number variation (CNV) and gene expression data to identify biomarkers or subtype of disease.

With advances in technology, generating high throughput genomics data has become affordable and popular for molecular profiling. As a result, many studies have generated multiple independent high throughput genomics data types on individual samples. In addition, large consortiums, such as The Cancer Genome Atlas (TCGA) and International Cancer Genome Consortium (ICGC), have generated several high throughput genomics data types for hundreds of sample on tens of cancer types, which are publicly available. Several methods have been used to integrate gene expression and CNV data. In general, these methods are based on three main approaches: 1-regression, 2-correlatin, and 3-module network [3]. Linear approaches, such as regression analysis or correlation analysis, do not work properly for heterogeneous data that their within-group variations are extremely high, such as ovarian cancer data. Further, in general, the relationship between gene expression and CNV is not linear. It has been shown that the module network analysis, which is a non-linear approach, performs well in identifying driver genes in cancer [1]. The key idea in the module network analysis, a form of Bayesian network analysis, is that similarly behaving variables can be grouped into “modules” and that the network can learn the same parents and parameters for each module, instead of each variable, as in a Bayesian network. A module can be defined as a set of random variables (in this application, a set of genes) that share a statistical model, for example, a set of genes that are co-expressed or co-regulated. In a Bayesian network, a different conditional probability distribution (CPD) is assigned to each random variable, whereas, in a module network, a CPD of a module is for all random variables in the same module. The main motivation for using module network analysis instead of regular Bayesian network analysis is that biological systems, similar to all complex systems, have too many variables but not enough data to robustly learn networks. In biological systems, we have thousands of genes but few samples. In addition, large networks are difficult to interpret, especially in biological systems. In addition, it is assumed that genes that are co-expressed are likely regulated in similar ways and might have the same drivers or regulators. In general, module network approach, which in this application is called gene module analysis [1, 4], is based on regression tree analysis (a form of probabilistic graphical models) to infer gene regulatory modules from gene expression data. This approach involves a search for the best fit tree, using a Bayesian scoring approach and the normal-gamma scoring function [4]. The learning procedure iteratively reassigns genes to the modules and searches for a tree with the highest score. This step specifies a set of modulator genes (roots of regression trees) that control and influence a module. The proposed method in [1], which uses CONEXIC software tool, employs gene expression values for training the tree while uses CNV genes as initial modulators. It uses only gene expression and CNV data and is not designed for comparative analysis, such as resistance versus responding.

The objective of this work is to develop a novel computation workflow to identify candidate biomarkers of drug resistance by integrating several genomics data types. Our goal is to identify a short list of genes as candidate drivers of resistance to anti-cancer drugs as a means to facilitate the biomarker discovery process. To accomplish this, we implemented a computational method based on gene module analysis, inspired by the work in [1], to integrate high throughput CNV, somatic mutation, and gene expression datasets. We utilized a data-driven approach to identify genes that stand out in resistant tumors but not in sensitive tumors and vice versa. To rank genes and select a subgroup of genes as inputs to the integrative method, we used the Earth Mover’s Distance (EMD) [5, 6] approach for differential analysis of gene expression and CNV data, as introduced in [7].

We applied the method to the challenging problem of identifying driver genes associated with drug response in ovarian cancer. Ovarian cancer is the deadliest of all the gynecologic cancers [8] and, according to the data, the mortality rates for ovarian cancer have not improved over the past 20 years. Combination of cytoreductive surgery and platinum-based chemotherapy is a routine treatment for almost all women diagnosed with ovarian cancer [9]. Mainly due to the emergence of chemotherapy resistance, many patients do not respond to the drug and ultimately succumb to the disease. Several studies have been conducted on drug resistance for ovarian cancer, however identifying robust predictors of chemotherapy response or resistance biomarkers remains challenging [10]. Data heterogeneity, which makes conventional approaches perform poorly, is one the major challenges in studying drug response for ovarian cancer. TCGA has collected detailed clinical records, including drug response information, and high-throughput genomics data for over 500 cases of ovarian serous cystadenocarcinoma. We used publicly available and clinically annotated gene expression, CNV, and somatic mutation datasets for ovarian cancer from TCGA. We showed that the developed method was able to identify highly related genes as candidate biomarkers of resistant.

## Methods

### Datasets and chemotherapy response

We used processed and normalized ovarian cancer genomics data as given by TCGA. We downloaded gene expression data from the Agilent 244 K Custom Gene Expression platform, CNV data from the Affymatrix Genome-Wide Human SNP Array 6.0 platform, and validated somatic mutation data from whole exome sequencing data obtained using Illumina Genome Analyzer DNA Sequencing. As of January 2015, genomics data of 570 patients with high-grade ovarian serous cystadenocarcinoma were available in TCGA. We examined the clinical data from these patients to identify eligible samples for determining cis-platinum chemotherapy response. To classify tumors into resistant and sensitive groups, we used a definition similar to one used by the TCGA group in [11]. Based on this definition, sensitive tumors have a platinum-free interval of six months or greater after the last primary treatment, do not have a sign of progression or recurrence, and have the follow-up interval of at least six months from the date of last primary platinum treatment; and resistant tumors are recurred within six months after the last treatment. Using the above definition, we identified 93 platinum-resistant and 231 platinum-sensitive primary tumors among the 570 patients, for which their gene expression, CNV, and somatic mutation data were available as well.

### Data selection using differential analysis

To select a subset of genes whose aberration/expression profiles were significantly different between sensitive and resistant tumors, we applied differential analyses to the gene expression, and CNV data of the two groups. One of the main challenges in analyzing ovarian cancer genomics data is data heterogeneity, where the within-group variation is high. This data heterogeneity makes conventional differential analysis methods perform poorly [7, 12–14].

**Fig. 1:**
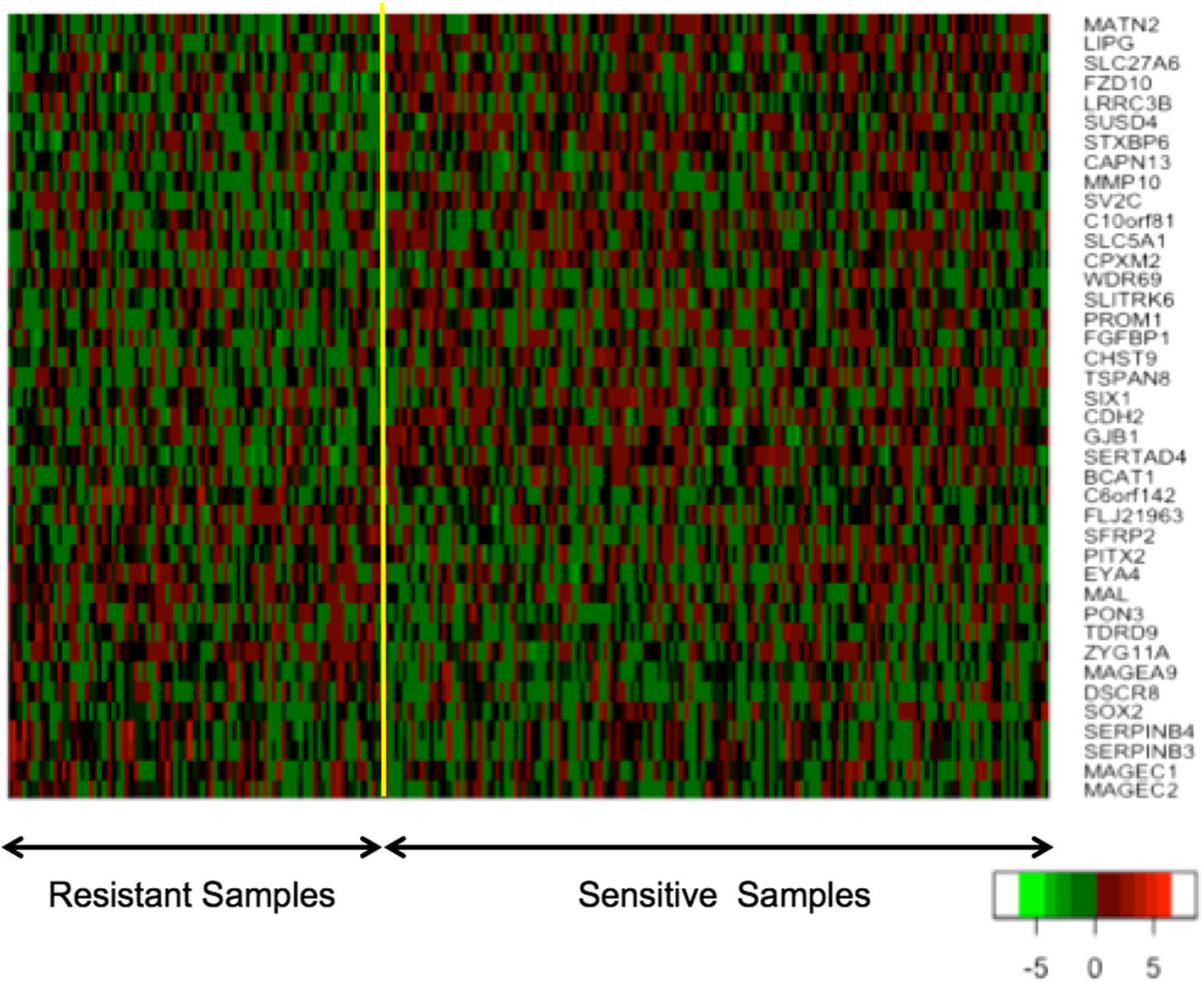
Heatmap of expression values of the 40 most significantly differentially expressed genes in TCGA ovarian cancer. Clustering method on expression values was used to generate the heatmap. Due to data heterogeneity, there are no clear clusters of genes for the resistant and sensitive groups

A heatmap of the most significantly differentially expressed genes (top 40 genes) in TCGA ovarian cancer data is shown in Fig. 1. As can be seen, expression profile is very heterogeneous, and there are no clear clusters of genes for the resistant and sensitive groups.

**Fig. 2:**
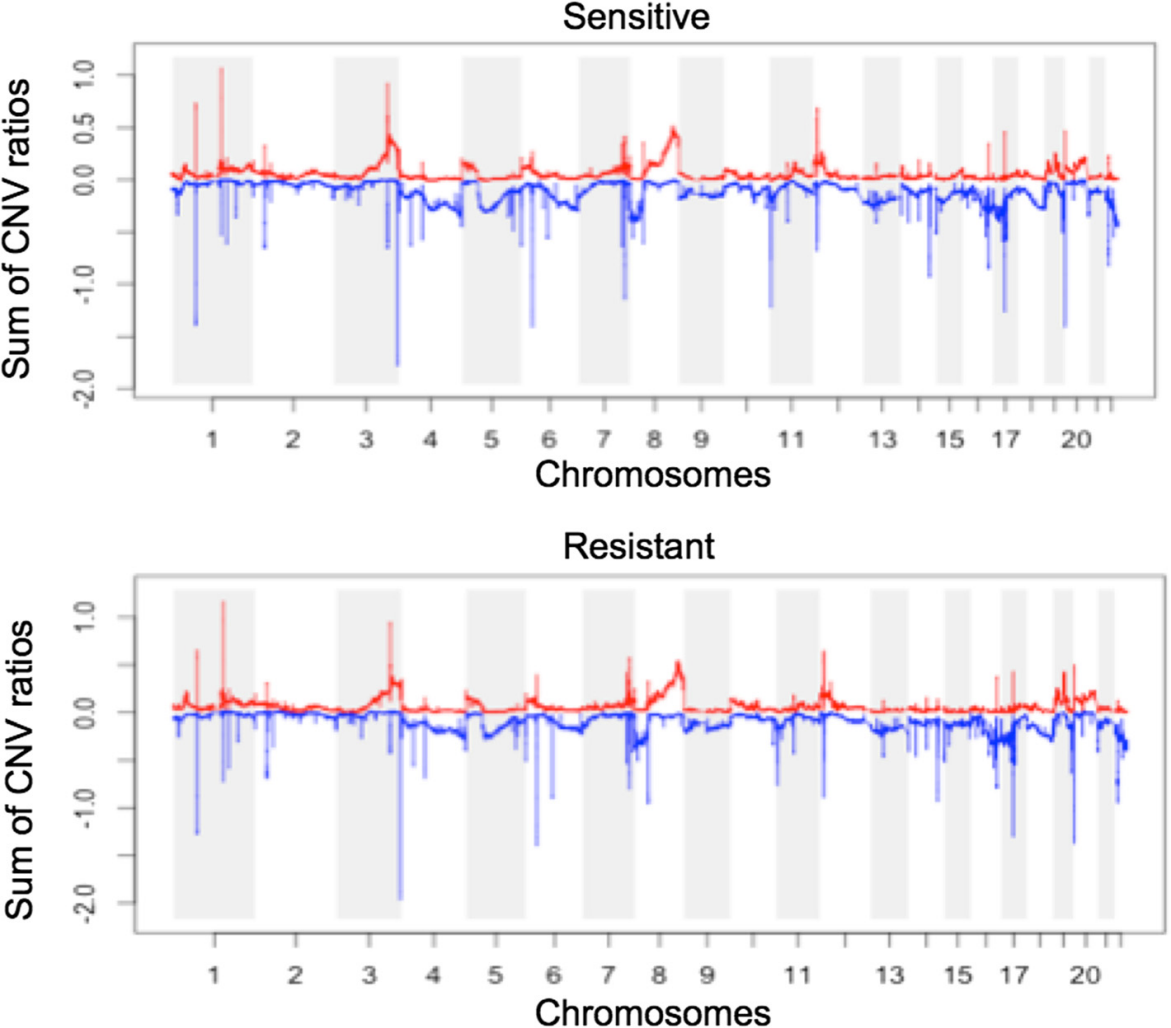
Cumulative CNV regions for resistant and sensitive TCGA ovarian cancer samples. CNV profiles of the two groups are very similar

The cumulative CNV profile of TCGA ovarian cancer samples for the two groups is shown in Fig. 2. As seen in the figure, the CNV profiles of the two groups are very similar as well, which makes it very difficult to identify CNV regions with unique amplification or deletion patterns that belong to only one group. Therefore, for differential gene expression analysis and to rank CNV genes, we used the EMD approach [5] as in [7], EMDomics, which is designed especially for heterogeneous data. We ranked genes based on their *q*-values and selected genes with a *q*-value < 0.1. For CNV data, first, we mapped genes to the CNV regions to obtain CNV genes; then, we used the EMD approach for the differential analysis and ranking of the CNV genes. We also calculated the frequency of amplification and deletion for each gene in the two groups and selected genes for which the difference between their frequencies is more than 20 %. We used a threshold of log2 copy number ratio of 0.3/−0.3 to call amplified/deleted genes.

**Fig. 3:**
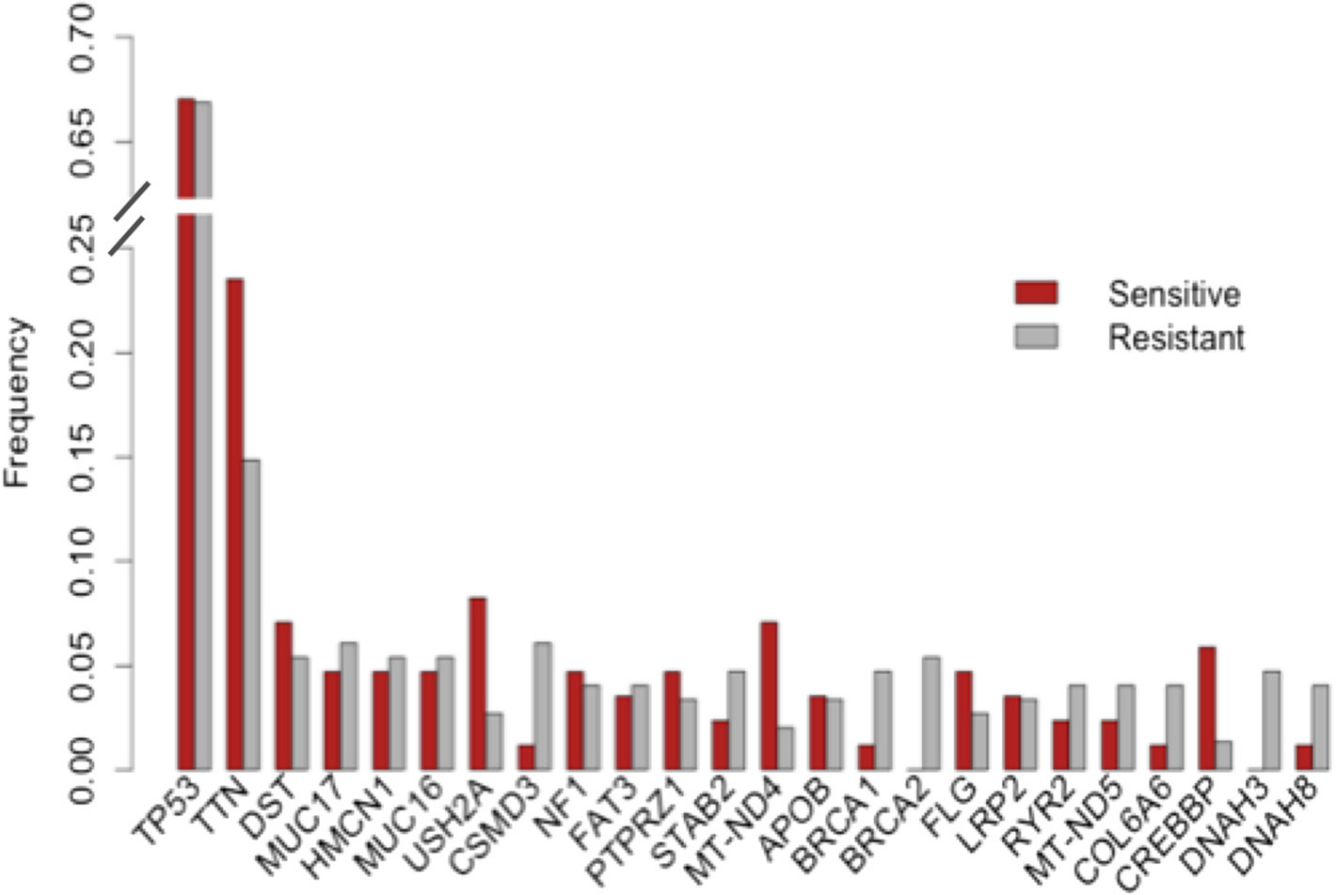
Somatic mutation frequencies for resistant and sensitive TCGA ovarian cancer samples. The frequency of mutations for most of the genes in the two groups is comparable

For somatic mutation data, we also calculated the frequency of mutations for all genes across the samples in each groups and selected the genes that were mutated in more than 2 % of the tumors. The top 25 genes with the highest frequency of mutation in the two groups are shown in Fig. 3.

**Fig. 4:**
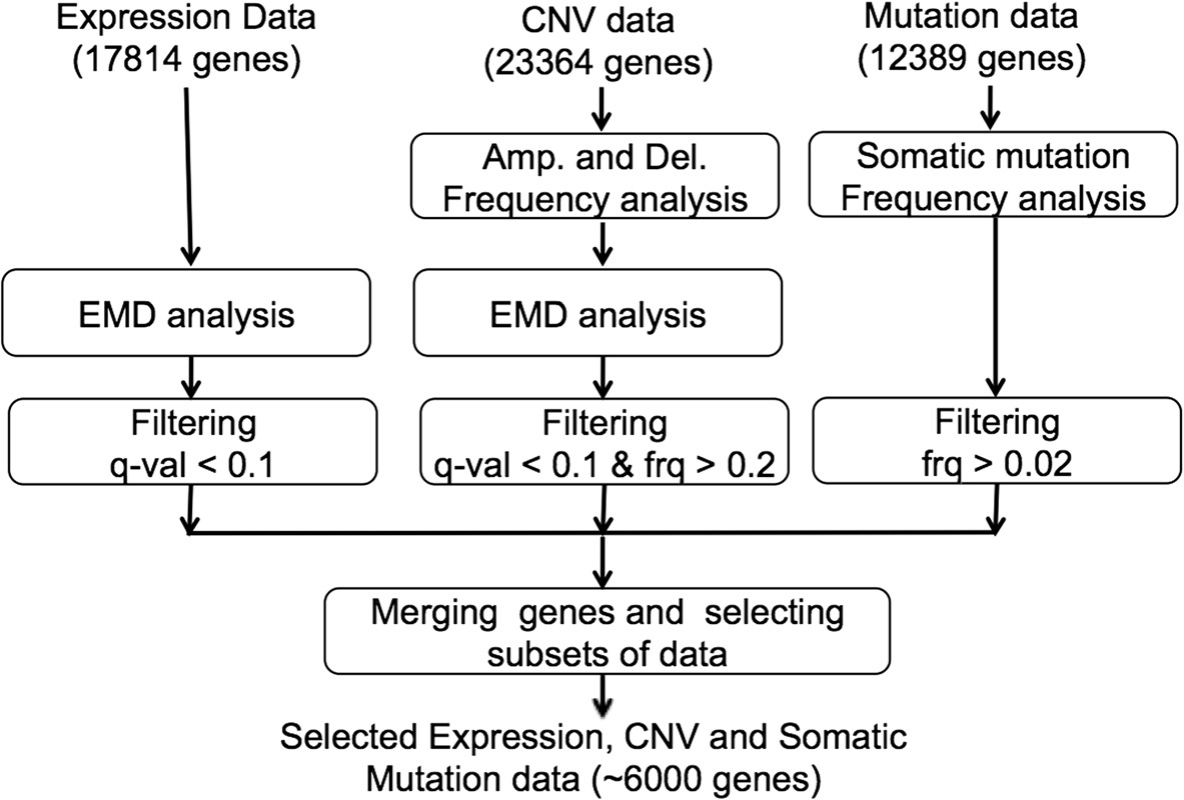
Schematic diagram of selecting a sub set of genes for integrative analysis

The overall procedure for selecting a subgroup of genes is shown in Fig. 4. The final list of genes for integrative analysis is a combination of genes for which gene expression values, CNV frequency rates, and CNV values differ significantly across the two conditions; and also somatic mutation rates are high. Genomics data of these genes were used in the integrative analysis, as explained below.

### Integrative analysis using gene module analysis

**Fig. 5:**
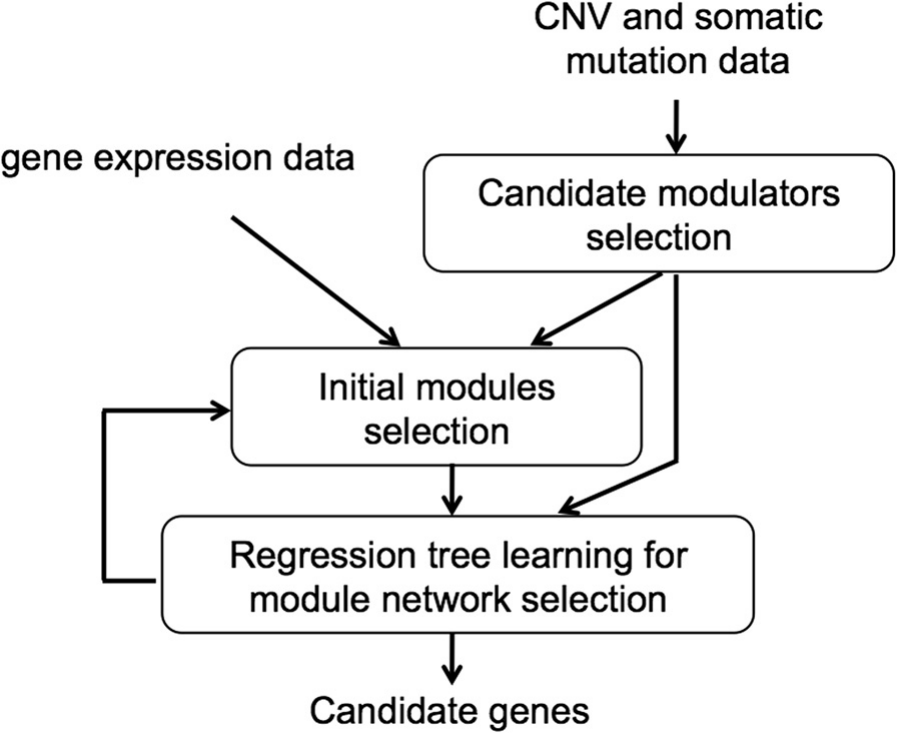
Schematic diagram of the module network analysis

We used module network procedure [1, 15] to integrate gene expression, CNV, and somatic mutation data. The overview of the overall integrative analysis is shown in Fig. 5. As can be seen in Fig. 5, the procedure takes as inputs these datasets and then produces a short list of genes as candidate biomarkers. As in [1], the analysis consists of three major parts: (1) selecting the candidate modulators (genes that regulate other genes in their module) from the list of aberrant genes, (2) obtaining the initial gene modules (groups of co-regulated genes) from the gene expression data, and (3) creating module networks [4] (Fig. 5).

Input datasets are for genes that are selected as described in the previous section. In addition, we selected genes as candidate modulators (in Part 1) if their CNVs and mutations frequencies were high across the tumors (mutation frequency >2 % and CNV frequency >70 %).

Before conducting the learning process, we created an initial association between candidate modulators and gene modules to aid the module network procedure. In this step, each candidate modulator is assigned to a cluster of genes to maximize the normal-gamma scoring function [1].

For gene module analysis we used CONEXIC software used in [1]. We modified the method in [1] such that it uses mutated and CNV genes as candidate modulators as well as uses these data for training the module networks.

### Comparative analysis, resistance versus sensitive

**Fig. 6:**
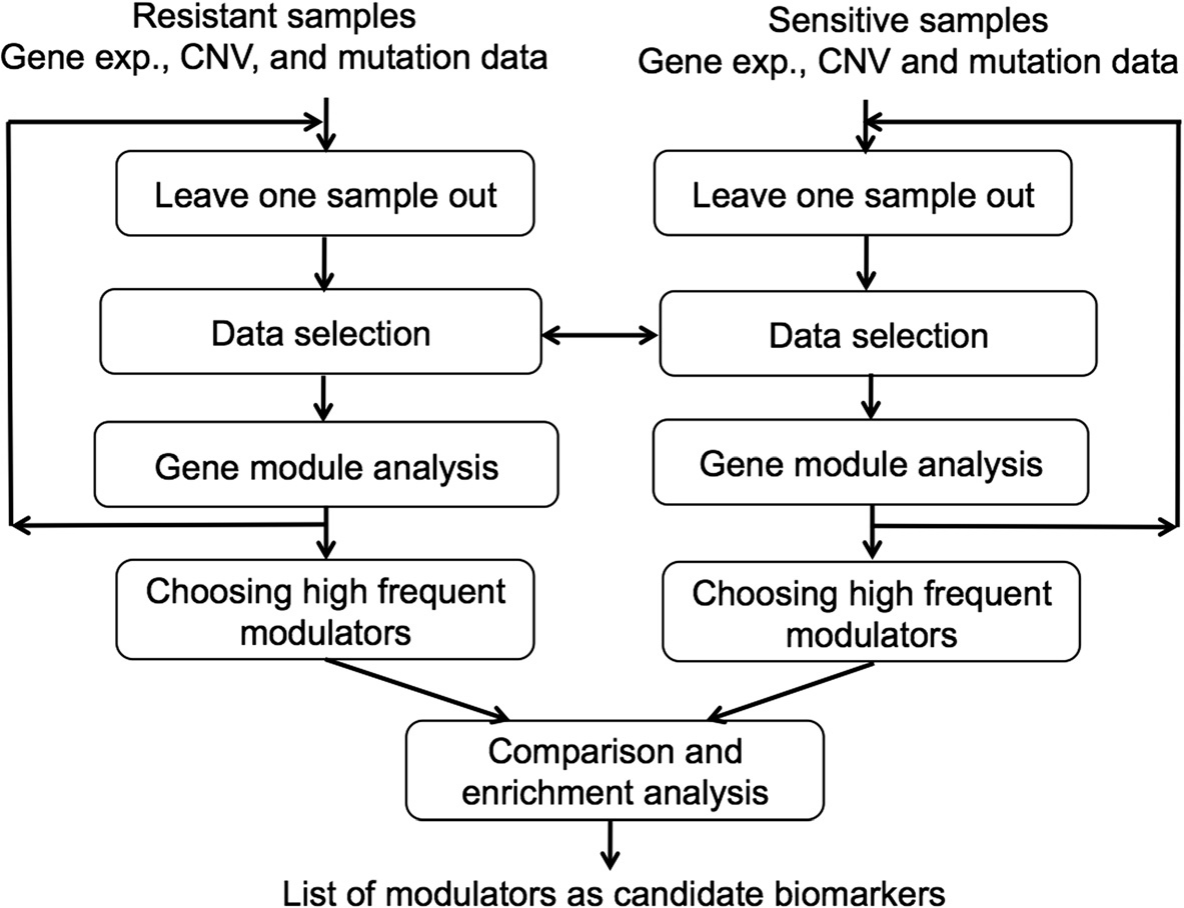
Schematic diagram of the comparative and the integrative analysis

We applied the above integrative analysis to the resistant and sensitive samples datasets separately and obtained their final modulator lists, as shown in Fig. 6. The expression data that were used to select the initial modules (Part 2 in the integrative procedure) are the same for the two conditions. The expression data are for the genes for which their gene expression, and CNV profiles differ significantly between the resistant and sensitive samples and their frequency of mutation is high, as explained in the previous section (Fig. 4). However, the candidate modulators (in Part 1 in the integrative procedure) are different for the two conditions. The candidate modulators are selected from the aberrant genes (CNV genes and mutated genes) that have a higher frequency of aberration for each condition. We compared the two final modulator lists and selected the genes that are modulators in the resistant group but not in the sensitive group and identified them as candidate resistant driver genes. The final result is a short list of candidate genes that are mutated (CNV and/or point mutation) and that regulate their gene modules.

### Cross-validation

To validate the final result of the gene module analysis, we used the leave-one-out approach for cross-validation. In each iteration, we took out one sample from the resistant group and one sample from the sensitive group. Then, we applied the selection and integration procedures. The overall block diagram of the method is shown in Fig. 6. When the cross-validation procedure was finished, we calculated the frequencies of the modulators for the resistant and sensitive samples and compared the lists of modulators for the two groups of samples. The high-frequency modulators in the resistant samples, but not those in the sensitive samples, are the final list of candidate biomarkers of resistance.

## Results and discussions

Our goal is to identify functional genes that harbor genomic aberrations, are responsible for drug resistance, and drive tumor progression. The assumption is that driver genes are among over/under-expressed genes that also have aberrations. We used gene expression data, CNV data, and somatic mutation data for 231 cis-platinum sensitive and 93 cis-platinum resistant ovarian cancer samples from the TCGA website. Sensitive and resistant samples are listed in Additional file 1. The gene expression data include expression level of 17,814 genes (Additional file 2). We obtained the CNV values of 23,364 genes for each sample by mapping CNV segments to genes (Additional file 3). The somatically mutated genes are listed in Additional file 4.

As explained, the integrative analysis is based on a statistical machine-learning approach, which involves an iterative search for the best model. To avoid random false positives, we selected features, or genes, that can differentiate resistant and sensitive groups significantly. Using all genes in the statistical integrative analysis will increase the rate of falsely identifying a driver gene by chance. Therefore, we selected a subset of genes whose aberration/mutation/expression profiles were significantly different between sensitive and resistant tumors, as described in the Methods section.

As seen in Fig. 3, the frequency of mutations for the genes in the two groups is comparable, including mutations in the Tp53, DST, and MUC17 genes. There are a few genes, such as BRCA1, BRCA2, and CSMD3, that show higher differences between mutation frequencies in the two groups. Therefore, for selecting somatically mutated genes we considered mutation frequencies for each group.

Combining the selected genes gave us a list of genes, which totaled 5808, for module network analysis. We also selected high-frequency mutated and CNV genes in each group as candidate modulators for the module network analysis. We compiled a list of 212 candidate modulators for the resistant tumors (Additional file 5) and a list of 555 candidate modulators for the sensitive tumors (Additional file 6).

We used the selected genes and candidate modulators to run the gene module analysis for the resistant and sensitive tumors. The output of the gene module analysis is a ranked list of high-scoring modulators that are both associated with differences in gene expression modules across samples and among aberrant genes in a significant number of these samples. The fact that the modulators are among aberrant genes (mutated, amplified, or deleted) indicates that they most likely control the expression of the genes in the corresponding modules. Because the modulators have aberrations in a significant number of tumors and they are the roots of the regression trees, it is reasonable to assume that a modulator can provide an advantage to the tumor and be considered as a driver. The procedure resulted in 306 modules with 32 modulators (Additional file 7) for the resistant samples and 580 modules with 67 modulators (Additional file 8) for the sensitive samples. Several modules shared the same modulators in each group of samples. Interestingly, the outputs of module network analysis for the resistant and sensitive groups shared only three genes, even though the same set of data was used as the input for training the network (Part 2 of the integrative analysis), but with different candidate modulators (Part 1 of the integrative analysis). In addition, the outputs of module network analysis from the resistant and sensitive groups are enriched for different biological pathways. This indicates the importance of the selection of candidate modulators.

**Fig. 7:**
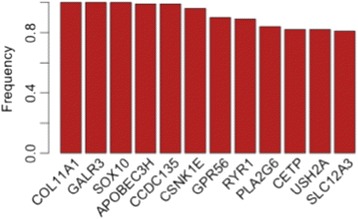
Signature of candidate biomarker of resistance to cis-platinum in ovarian cancer, using TCGA ovarian cancer data

We applied enrichment analysis, using GeneGo’s MetaCore pathways analysis software tool (http://thomsonreuters.com/metacore/), to the outputs of the module network analysis. Because the gene list contains 32 genes, enrichment analysis provides a few biological pathways with a *q*-value < 0.05. However, these genes are involved in important pathways, such as WNT signaling, DNA damage, and cell cycle pathways. The top 20 pathways are listed in Additional file 9. Network analysis of these 32 genes indicates the connection of these genes with biological networks that have important hubs, such as P53 (*p*-Value = 9.03e-12), a tumor suppressor gene which is associated with a variety of human cancers (Additional file 10: Figure S1); c-Myc (*p*-Value = 3.06e-9), which plays a role in cell cycle progression, apoptosis, and cellular transformation and is associated with a variety of tumors (Additional file 10: Figure S2); DNMT1 (*p*-Value = 1.65e-5), which is associated with certain human tumors and developmental abnormalities (Additional file 10: Figure S3.); and ESR1 (*p*-Value = 7.92e-7), which has important role in DNA binding and is associated with several cancer types including ovarian cancer [16] (Additional file 10: Figure S4). The list of the biological significant networks is provided in Additional file 11. After cross-validation (leave-one-out cross validation), we obtained a short list of genes that appeared frequently in both the resistant and sensitive samples. Twelve genes appeared more than 80 % of the time as modulators of the resistant samples but not the sensitive samples, as shown in Fig. 7. This short list of genes indicates very biologically meaningful results: All but one of these genes have been associated with several kinds of neoplasms and/or carcinomas: GPR56 has a role as an inhibitor of tumor progression [17]; GALR3 has been shown to be present in certain liver and brain tumors [18, 19]; APOBEC3H is associated with the immune system GO term and has been associated with lung and kidney carcinoma; CCDC135 is associated with the cell differentiation GO term and a variety of cancers, such as breast cancer and colorectal cancer [20, 21]; SOX10 is associated with the cell development and differentiation GO terms and several cancer types, such as pancreatic and prostatic cancer [22, 23]; PLA2G6 is associated with several cancer types, such as colorectal cancer [24]; USH2A is also associated with several cancer types, including ovarian cancer and breast cancer [20, 24]; and CSNK1E is involved in the Development_WNT signaling and DNA replication and repair pathways and is associated with variety of cancer types, including ovarian cancer and breast cancer [25–28]. Interestingly, CSNK1E, USH2A, and COL11A1 [29, 30] have been associated with poor survival in ovarian cancer. Further, COL11A1 is reported as a cis-platinum resistant biomarker for epithelial ovarian carcinoma cell lines [30–32]. Further experimental validation of the candidates’ pathways and/or genes in the wet laboratory in paired platinum-sensitive and -resistant ovarian cancer cell lines is required to demonstrate their functional involvement in the platinum-resistant phenotype, which is outside the scope of this work.

## Conclusions

With advances in sequencing technologies and the availability of an increasing amount of high throughput genomics data, the use of advanced computational methods to analyze, integrate, and mine the huge amount of genomics data is an absolute necessity. In particular, the complexity and heterogeneity of cancer genomics data and the level of noise in these data make the use of computational methods and statistical machine-learning approaches essential to understanding the key biological functions. In addition, with availability of genome-wide genomics, transcriptomics, and epigenomics data, unbiased data driven approaches can present an opportunity for generating new hypotheses and discoveries.

The main goal of this study was to generate a biologically meaningful short list of genes as candidate drivers of drug resistance by mining genomics data and using computational methods. We presented a nonlinear statistical approach to integrate gene expression, copy number aberration, and somatic mutation data to identify candidate drivers of resistance. First, to reduce random false positives in the identification of driver genes, we selected a subset of genes that differentiate the two groups of samples, taking into consideration gene expression, CNV, and somatic mutation data. For the differential analysis of gene expression and copy number data, we used the EMD approach [7] that is designed especially for highly heterogeneous data. We used mutation frequency for each gene to select somatically mutated genes. The integrative analysis, which is based on the module network analysis and uses the gene module analysis method as in [1], was run separately on the resistant and sensitive samples to obtain driver genes for each condition. In this way, we identified aberrant genes that have the most influence on the groups of co-regulated genes. Finally, we used a leave-one-out approach for cross-validation of the results of the integrative analysis and compared the results for the sensitive and resistant groups to identify driver genes.

We applied this method to the challenging problem of identifying candidate biomarkers of drug resistance in ovarian cancer. We chose ovarian cancer because ovarian cancer genomics data are very heterogeneous, and conventional methods for analyzing resistant and sensitive samples perform poorly. In addition, TCGA provides rich clinical data on drug response for ovarian cancer samples, in addition to their genomics data, which are publicly available. The final result is a short list of biologically meaningful genes that are frequently identified as modulators or drivers in the resistant samples but not in the sensitive samples. For example, COL11A1 is identified as a candidate biomarker that has been reported as a cis-platinum resistant biomarker for epithelial ovarian carcinoma in several recent studies [30–32].

The strongest aspects of the method are that it is a data-driven and unbiased approach that does not make any assumption about the distributions of the data. In addition, the integrative method is nonlinear and does not make the assumption of a linear relationship between gene expression values and genomics aberration values. Further, the module network analysis is computationally feasible for large genomics datasets. Nevertheless, there is “no free lunch” in computational and statistical analysis. This method is computationally expensive due to iteration in training the model and searching for the best model as well as due to the cross-validation procedure. In addition, because it is a statistical machine-learning method, it works better when there are more samples. For studies with only a few samples in each condition, this method cannot perform well.

To conclude, we developed a novel data-driven approach based on statistical machine-learning analysis to uncover candidate biomarkers of drug resistance, and we showed that that method can yield a short list of biologically meaningful genes that can be used to facilitate the biomarker discovery process.

## Abbreviations

TCGA, The Cancer Genome Atlas; CNV, copy number variation; EMD, Earth Mover’s Distance; CPD, conditional probability distribution

## Additional files

Additional file 1: List of ovarian cancer TCGA samples that are used in this study. (TXT 8 kb) Additional file 2: Expression data. (TXT 8662 kb) Additional file 3: CNV data. (TXT 14949 kb) Additional file 4: Somatic mutation data. (TXT 260 kb) Additional file 5: List of initial modulators for the resistant group. (TXT 1 kb) Additional file 6: List of initial modulators for the sensitive group. (TXT 3 kb) Additional file 7: Modulator list for the resistant group. (TXT 206 bytes) Additional file 8: Modulator list for the sensitive group. (TXT 428 bytes) Additional file 9: Top 20 pathways enriched by the resistant modulators. (XLSX 43 kb) Additional file 10: Plots of enriched biological networks for resistant modulators. (PDF 3200 kb) Additional file 11: List of enriched biological networks for resistant modulators. (XLS 39 kb)
