# Supplementary material for: Identifying candidate drivers of drug response in heterogeneous cancer by mining high throughput genomics data
Source: BMC Genomics. 2016 Aug 15;17:638. doi: 10.1186/s12864-016-2942-5 (PMC4986197; doi:10.1186/s12864-016-2942-5)
Supplement: Additional file 10: — Plots of enriched biological networks for resistant modulators. (PDF 3200 kb) [file 12864_2016_2942_MOESM10_ESM.pdf]

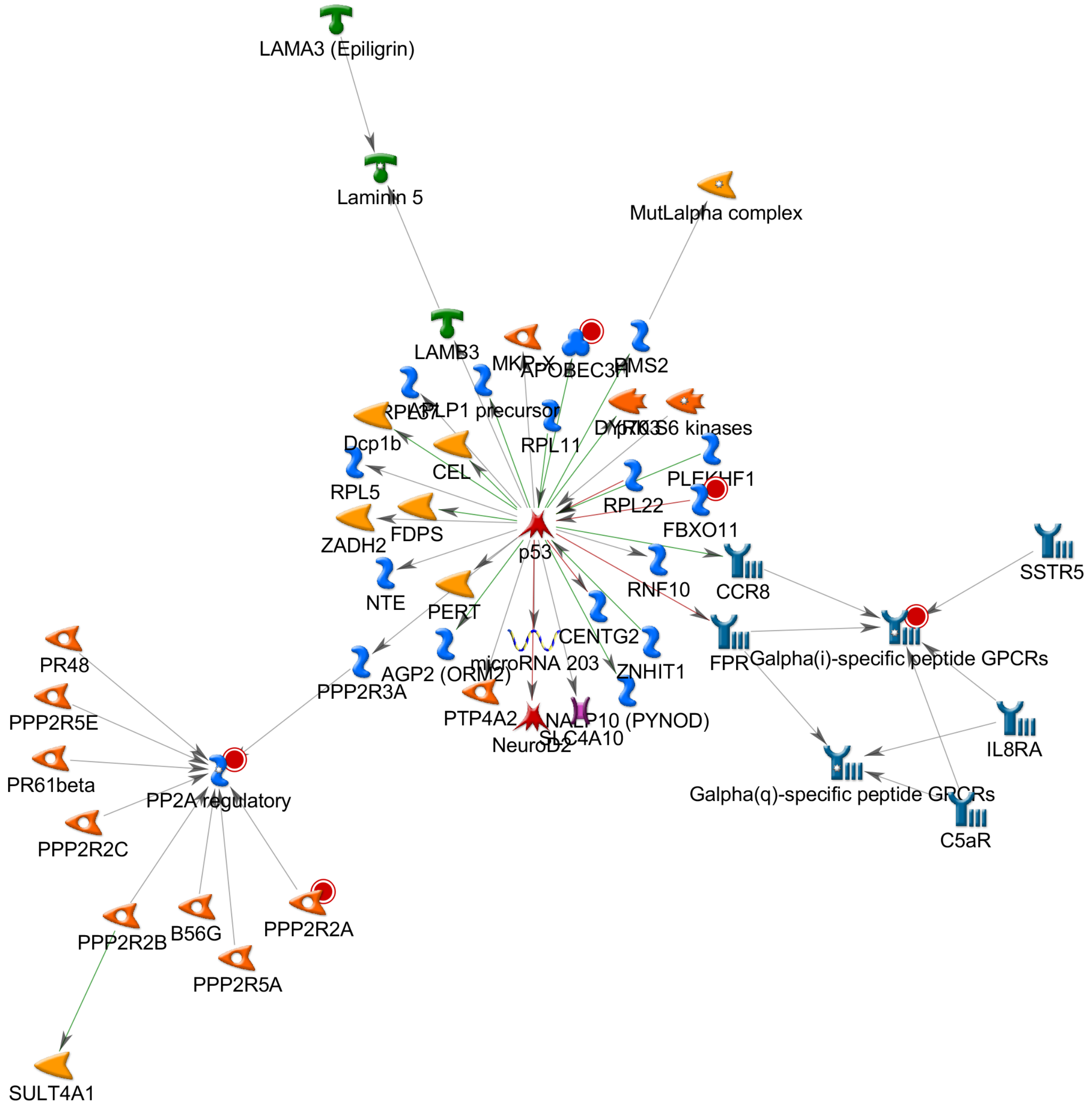

Fig. S1. APOBEC3H, FBXO11, p53, RPL37, SLC4A10 Network. Candidate driver genes are marked with red circles.

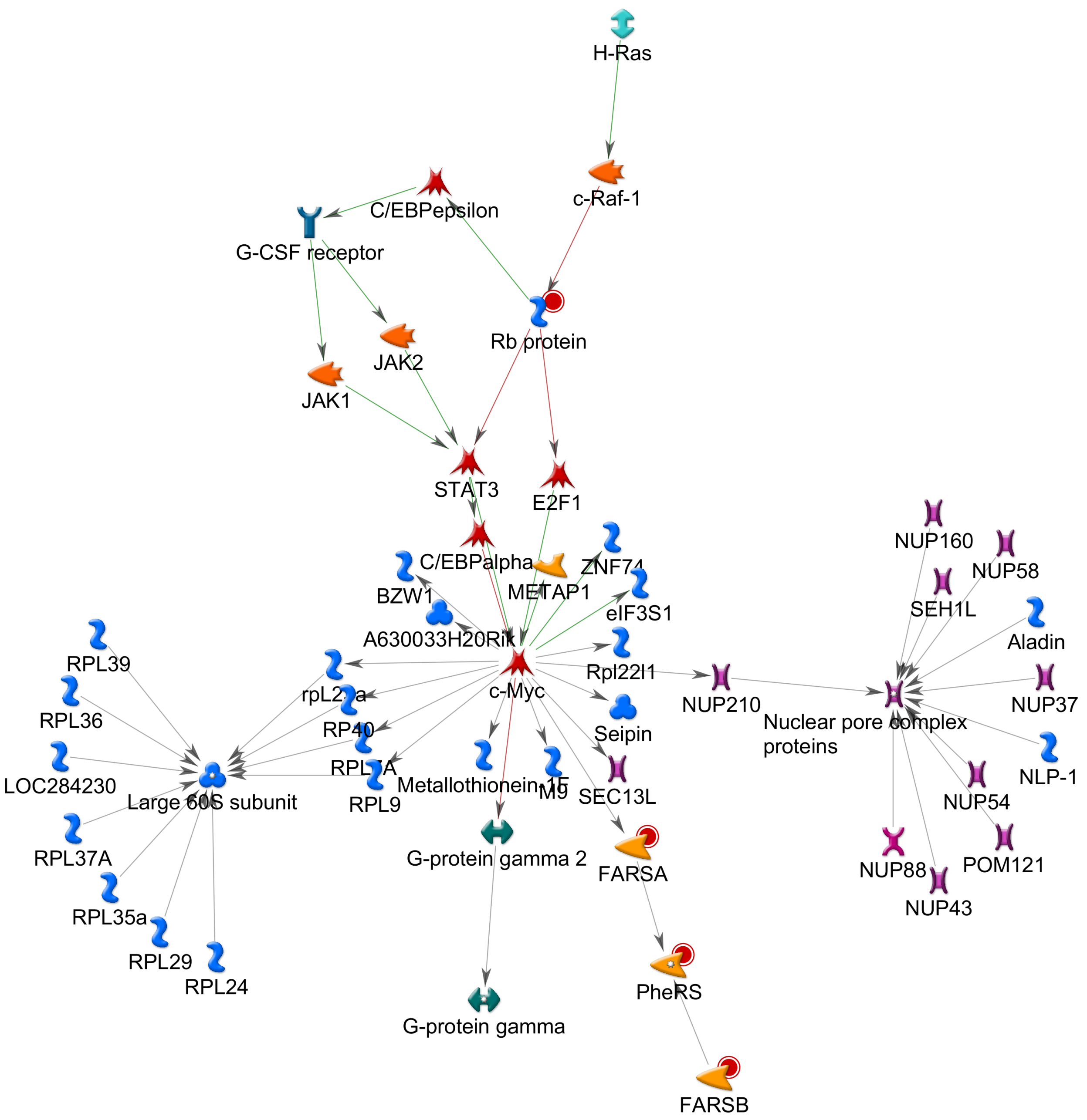

Fig. S2. Rb protein, FARSA, c-Myc, STAT3, G-CSF receptor Network. Candidate driver genes are marked with red circles.

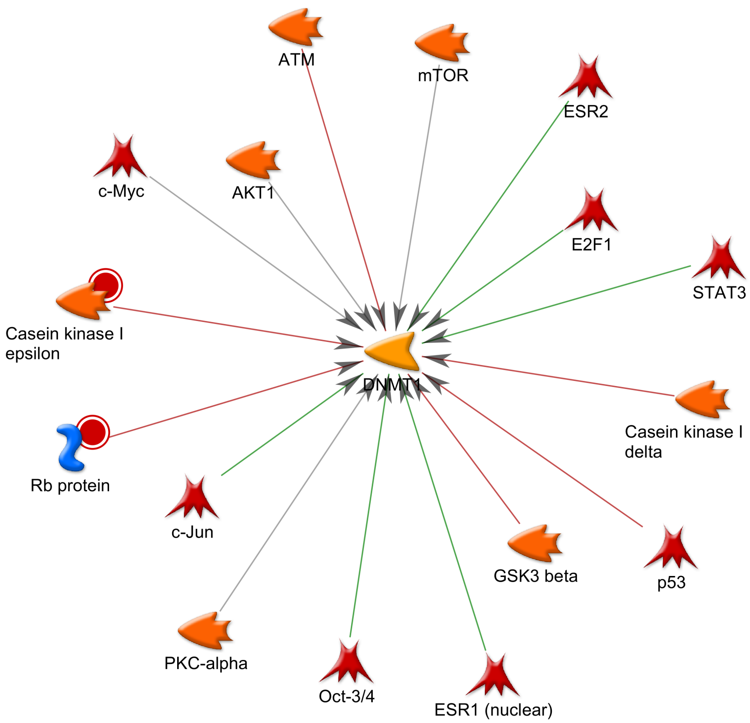

Fig. S3. Casein kinase I epsilon, Rb protein, DNMT1, ESR1 (nuclear), mTOR Network. Candidate driver genes are marked with red circles.

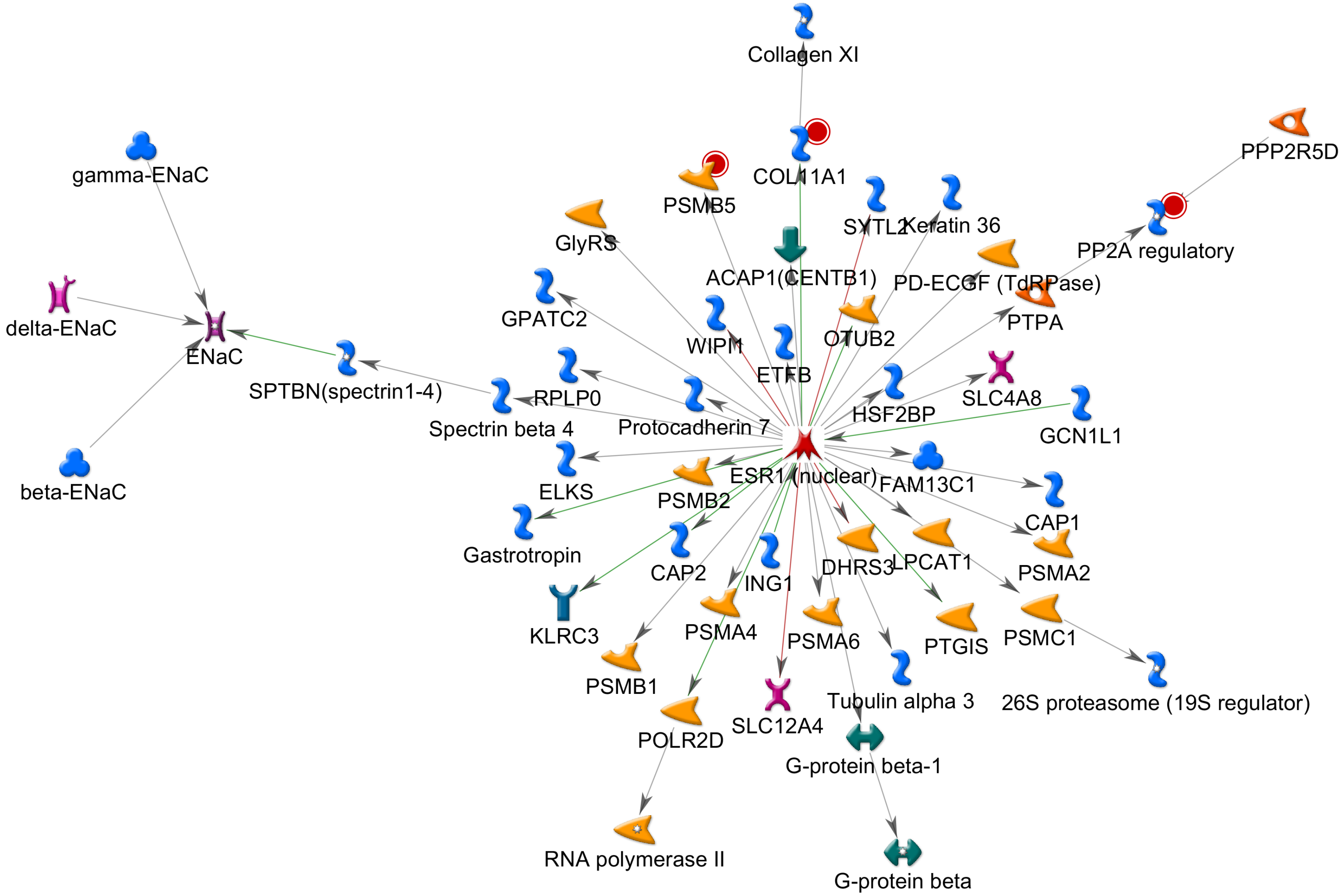

Fig. S4. COL11A1, PSMB5, ESR1 (nuclear), PSMC1, PTPA Network. Candidate driver genes are marked with red circles.
